# HEALTH SYSTEM ADOPTION OF COMMUNITY-BASED EXERCISE FOR OLDER ADULTS TO ADDRESS LONELINESS AND SOCIAL ISOLATION

**DOI:** 10.1093/geroni/igae098.1069

**Published:** 2024-12-31

**Authors:** Allison Mays, Alexander Lawton, Melissa Mandujano, Leilani Martinez, Nathalie Guevara, Jonathan Vickburg, Sonja Rosen

**Affiliations:** Cedars-Sinai Medical Center, Los Angeles, California, United States; Cedars-Sinai Medical Center, Los Angeles, California, United States; Cedars-Sinai Medical Center, Los Angeles, California, United States; Cedars-Sinai Medical Center, Los Angeles, Colorado, United States; Cedars-Sinai Medical Center, Los Angeles, California, United States; Cedars-Sinai Medical Center, Los Angeles, California, United States; Cedars-Sinai Medical Center, Los Angeles, California, United States

## Abstract

Transitioning the delivery of evidence-based programs (EBPs) for older adults from research to community-based settings potentially alters programs’ reach. We compared the demographics of our research participants to our referred community participants after the program’s adoption by Cedars-Sinai, Community Health Improvement (CHI). Research participants enrolled in Falls Prevention EBPs reported decreased levels of loneliness and social isolation and were referred by physicians, social workers, and pharmacists using an order in the electronic medical record (EMR). Clinicians referred 1355 in-person participants (July 2017-March 2020) and 283 virtual (April 2020- May 2021). Enrolled research participants (in-person (n=382) /virtual (n=214)) had an average age of 76.6 / 76.5 years; were 83.4% / 82.5 % female; and non-Hispanic white 42 % / 70.4%; and non-Hispanic Black 43.5% / 8.2%. CHI retained the relationship with the non-profit partner who ran the study’s EBPs and the EMR order remained active and received 391 referrals (July 2021 – March 2024). Referred patients had an average age of 79.5 years, 79.3% female, 59.5% non-Hispanic white, 21.5% Black, 8.2% Hispanic, 5.9% Asian, 3.1% Other; 68.5% had Medicare and 31.7% Medicare Advantage. Referral orders were associated with diagnoses for preventative visits (18.7%), falls (15.6%), and cognitive changes (13.6%). Compared to research participants, community referrals included those with cognitive impairment, were older, more male, and more racially diverse than virtual study participants. Community implementation of EBPs may reach a more diverse population than research participants and successful transition of programs can be facilitated through preserving prior community partnerships and referral pathways.

